# Receptive Vocabulary Outcomes in Children with Cochlear Implants with and Without Additional Difficulties: A Multicenter Cross-Sectional Analysis

**DOI:** 10.3390/audiolres16020053

**Published:** 2026-04-02

**Authors:** Beauty Hariz, Latifa Alkoheji, Mariam Alsaeed, Amany Tahon, Shahad Alhammad, Maram Alhedaithy, Sara Ali AlKhamiss, Hasna’a Shathan, Toga Alharbi, Salam Orabi, Sabine El-Deek, Per Cayé-Thomasen, Lone Percy-Smith

**Affiliations:** 1 Copenhagen Hearing and Balance Center, Department of Otorhinolaryngology, Head and Neck Surgery and Audiology, Copenhagen University Hospital Rigshospitalet, 2100 Copenhagen, Denmark; per.caye-thomasen.01@regionh.dk (P.C.-T.); lone.percy-smith@regionh.dk (L.P.-S.); 2 ENT Department, Salmaniya Medical Complex, Manama 2904, Bahrain; latifa_alkooheji@msn.com; 3 Sheikh Salem Al-Ali Centre for Hearing and Speech, Shuwaikh Industrial, Safat 13041, Kuwait; momoslp2005@gmail.com; 4 Audiology & AVT Department, Cochlear Implant Center, King Saud Medical City, Riyadh 12746, Saudi Arabia; amousa@rfhc.gov.sa (A.T.); shalhammad@rfhc.gov.sa (S.A.); msalhedaithy@ksmc.med.sa (M.A.); 5 King Abdullah Ear Specialist Center, King Saud University Medical City, Riyadh 12629, Saudi Arabia; saraalkhamiss@gmail.com; 6 Cochlear Arabia Regional Headquarters, Riyadh 12361, Saudi Arabia; hshadhan@cochlear.com (H.S.); salam.orabi@hotmail.com (S.O.); 7 Audiology & Speech Department, UHUD General Hospital, Medina 42354, Saudi Arabia; togaa988@gmail.com; 8 ENT Department, Al Jalila Children’s Specialty Hospital, Dubai P.O. Box 7662, United Arab Emirates; sabinedeek@gmail.com

**Keywords:** Pediatric Cochlear Implant, receptive vocabulary, additional difficulties

## Abstract

**Background/Objectives:** Receptive vocabulary is essential for children’s language, academic, and cognitive development. While cochlear implants (CIs) help children with severe to profound hearing loss develop spoken language, their vocabulary skills often fall behind their typical hearing (TH) peers, although early implantation and auditory-verbal therapy (AVT) can help narrow this gap. Children with CIs and other developmental difficulties face additional challenges, but can still progress, with outcomes depending on the disabilities’ type and severity. Limited research exists on Arabic-speaking children with CIs, where cultural factors may delay intervention, and outcomes vary widely. It remains unclear how well these children develop receptive vocabulary compared to hearing peers and which factors influence their progress. **Methodology:** A multicenter, cross-sectional study in six GCC hospitals compared 103 children with CIs to a control group of 94 children with TH. Children with CIs were divided into those with and without additional difficulties. Receptive vocabulary was evaluated utilizing the Peabody Picture Vocabulary Test, Fifth Edition. **Results:** Children with CIs in the GCC scored lower (mean 89.5; SD = 20.5) than the TH control group (mean 104; SD = 16.8). Children with CIs without additional difficulties (mean 97.7; SD = 18.8) scored similarly to TH, while children with CIs and additional difficulties scored significantly lower (mean 76.7; SD = 15). Age at switch-on and presence of additional difficulties significantly affected receptive vocabulary outcomes. **Conclusions:** Children with CI who have no additional disabilities can reach receptive vocabulary levels similar to typical hearing peers, while those with extra difficulties show very diverse outcomes and continue to face challenges.

## 1. Introduction

Receptive vocabulary refers to the ability to understand words and is vital in language development, particularly for young children [1]. Perry et al. demonstrate that vocabulary predicts expressive and receptive progress over time [2]. It facilitates the ability to follow instructions [3], comprehend narratives [4], and communicate effectively. This skill is closely linked to grammar, reading comprehension, and academic achievement, making it a key indicator of overall language proficiency [5]. Additionally, receptive vocabulary supports the ability to acquire new knowledge [6]. Children who acquire a larger receptive vocabulary early on will likely develop more advanced grammar and expressive language skills later [5,7]. Receptive vocabulary also forms the foundation for literacy and math skills [1,8], and it is not separable from overall cognitive function. Working memory, attention, and processing speed all contribute to the pace and efficiency at which children can learn new words [9]. Substantial evidence supports the connection between vocabulary and spoken language, academic achievement, executive function, and cognition [10]. Therefore, tracking receptive vocabulary growth gives a glimpse into a child’s future social, cognitive, and academic potential.

Early sound exposure occurs during critical periods of language development when the brain is most receptive to sound and language learning [11], and early speech perception is strongly linked to early vocabulary growth [12]. For children with severe to profound hearing loss (HL), cochlear implants (CIs) may provide auditory access that supports listening and spoken language development [13,14]. For these children, receptive vocabulary becomes especially important as they work toward developing spoken language and accessing advanced literacy and academic skills [15], which plays an important role in shaping their language profile [4]. Although early implantation, consistent device use, along with speech and language therapy focused on listening and spoken language, have been shown to often contribute to improved outcomes, research shows that children with CIs often have lower receptive vocabulary levels compared to typically hearing children [16,17]. However, other studies have also found that children with CIs can achieve vocabulary skills within the norm when favorable conditions are present [18,19]. Further studies indicate that children who are implanted early tend to have more extensive receptive vocabularies and language skills than those implanted later and are closer to typically hearing children [20]. Still, not all early-implanted children succeed equally; some research has shown variable results in receptive vocabulary, with some achieving normative levels while others are significantly delayed [14]. The choice of communication approach for children with severe to profound hearing loss remains a topic of ongoing discussion in the literature. While bilingual approaches incorporating visual elements have historically been advocated, advances in early identification and cochlear implant technology have increased the potential for the development of listening and spoken language in many children [21]. The present study specifically focuses on spoken language outcomes, specifically receptive vocabulary, in children with CIs.

The difficulties in developing receptive vocabulary become greater when children with CIs also face other developmental challenges. The percentage of children with HL and additional disabilities varies in the literature, but several studies indicate that about 30–40% have coexisting disabilities [22,23,24]. Still, approximately half of these children improve in their speech awareness and receptive language [25]. This progress is closely linked to the type and severity of their other difficulties [23]. Percy-Smith et al. have found that children with additional disabilities showed slower but nonetheless positive growth in their receptive vocabulary over six years [19].

Research involving Arabic-speaking children with CI is limited, but emerging evidence aligns with global trends. Children are often diagnosed and implanted later than in other countries [26], as cultural attitudes toward disability and the stigma around HL can delay seeking the appropriate intervention [26,27]. The majority of the existing research has concentrated on speech perception, speech production, and speech intelligibility [27] and shows a high and significant level of interindividual variability [28].

Conditions across the Arab world vary, but the countries of the Gulf Cooperation Council (GCC) are united by social, religious, cultural, and economic characteristics, as well as by health challenges [26,29,30]. There is very limited research, to our knowledge, on whether children with CIs in the GCC develop a receptive vocabulary that allows them to acquire complex language and literacy skills later on and whether their levels are comparable to those of their hearing peers. It is also unclear what specific factors influence their progress and how challenges affect the development of their receptive vocabulary.

### Aim of Study

The overall aim of our study was to map the level of receptive vocabulary, as an indicator of spoken language development, among Arabic-speaking children in a multicenter study across the GCC countries. A secondary aim was to compare children with CI with and without additional disabilities, and to compare each group to a representative group of children with typical hearing.

## 2. Materials and Methods

### 2.1. Study Design

This multicenter study is an explorative, descriptive, cross-sectional, and comparative research conducted across six hospitals in four GCC countries: Saudi Arabia, Kuwait, the United Arab Emirates, and Bahrain. The study adopted a cross-sectional design, providing a snapshot of children with CIs at the time of testing and assessment, comparing receptive vocabulary outcomes between children with CIs (with and without ADs) and a TH control group at a single time point. All children attended testing with one or two accompanying parents. Qualified speech and language professionals from six hospitals, all experienced in evaluating children with HL and extensively trained in listening and spoken language development, focused on the 10 auditory-verbal therapy (AVT) principles in their practice and carried out the assessment. All participating centers followed the same testing protocol, including administration procedures and instructions based on the PPVT-5 manual. Minor variations were limited to dialectal adaptations. An independent speech and language pathologist, who was not involved in the testing, scored the tests. Scoring was performed according to the PPVT-5 manual [31]. We collected demographic details such as age at diagnosis, prior use of hearing aids, age at implantation, etiological factors, additional disabilities, consanguinity between parents, maternal employment status, and parents’ educational levels from parental questionnaires and the children’s medical records.

### 2.2. Description of Test Material

This study investigates how receptive vocabulary develops in children using the Peabody Picture Vocabulary Test 5th edition (PPVT-5) [31]. The PPVT-5 is a widely used norm-referenced test of receptive vocabulary. It has a mean standard score of 100 with a standard deviation (SD) of 15 (normative range: 85–115). The test is available for children from two years and six months up to adulthood. During testing, children are asked to point to one of four pictures representing the word spoken by the therapist.

Since the test is not standardized in Arabic, an Arabic-adapted version of PPVT-5 was translated for this study by two speech-language pathologists. Then, at least two speech therapists from each GCC country, who were not involved in the translation, reviewed and adjusted the standardized Arabic words to ensure they fit the dialect spoken in their respective countries. The adapted Arabic translations were then backtranslated into English by two bilingual (English/Arabic) individuals to verify accuracy and consistency.

To address the absence of standardized Arabic norms, the PPVT-5 standard scores were interpreted with caution, and a typical hearing (TH) control group was also created to compare its scores with both the norm group and the CI group for context and as a reference alongside the original normative data.

### 2.3. Participants

The participants were Arab children diagnosed with prelingual bilateral severe to profound sensorineural HL and implanted, before the age of three and a half years, at one of the participating hospitals. Their hearing age was at least 2 years, and they were actively receiving or had previously received AVT services at the selected hospitals. We accessed the patient data from the medical records after obtaining consent from the children’s legal guardians. The participants (n = 103) were allocated to one of two cohort groups: 63 children with CI without additional difficulties (CI − AD) (61.2%) and 40 children with CI and additional difficulties (CI + AD) (38.8%). Some children were diagnosed with one or more additional difficulties (ADs). ADs were identified based on documented clinical diagnoses in the children’s medical records, established by multidisciplinary teams. The classification relied on existing clinical documentation rather than on standardized assessment protocol conducted within the study. Demographics and descriptive characteristics of participants are presented in Table 1 and Table 2.

A control group of 94 children with TH was included for comparison. This group was from the 4 countries included in our research. 53.2% (n = 50) were from KSA, 30.9% (n = 29) from Bahrain, 10.9% (n = 10) from Kuwait, and 5.3% (n = 5) from UAE. Children were 51.1% male and 48.9% female. Their age varied between 2.5 and 15 years, with a mean of 7.93 years and a SD of 3.66. At the time of the testing, those children did not have any diagnosed ADs. They all have done the PPVT test, and their standard score varied between 71 and 145, with a mean of 103.29 and a SD of 16.57.

### 2.4. Ethical Considerations and Approvals

This study is based on a cross-sectional comparative study design. No intervention was conducted. Access to patient data in medical records was strictly contingent upon obtaining written consent from the children’s legal guardians. Measures were implemented to protect the privacy of participants’ data and ensure confidentiality. All personal data related to children and families was anonymized.

The ethics committees for scientific research in the participating countries/hospitals have approved the study:

- The Institutional Review Board of King Saud Medical City No. H1RI-09-May23-03, accessed on 26 September 2023.
- The Research Department of the Ministry Of Health in Kuwait, No. 2297-2023, accessed on 29 March 2023.
- The Research Committee for Government Hospitals in Bahrain, No. 98260923, accessed on 26 September 2023.
- Institutional Review Board at King Saud University Project No. E-24-8600, accessed on 3 April 2024.
- The Institutional Review Board of the Mohammed Bin Rashid University for Medicine and Health Sciences in Dubai: MBRU IRB-2023-250, accessed on 10 January 2024.

### 2.5. Data Analysis

The statistical analysis was performed using IBM SPSS Statistics version 27. Descriptive statistics were presented as frequencies and percentages for categorical variables, and as means, medians, standard deviations, minimums, and maximums for continuous variables.

The Kolmogorov–Smirnov and Shapiro–Wilk tests were conducted to evaluate the normal distribution of the data. A *p*-value less than 0.05 indicated a significant deviation from normality. When the assumption of normality was not met, non-parametric tests were applied. Specifically, the Mann–Whitney U test was used for comparisons between two groups, while the Kruskal–Wallis test was used for comparisons involving more than two groups. The presence of ADs was addressed through subgroup comparisons (CI − AD vs CI + AD) rather than inclusion as a covariate in multivariate models.

In addition, cross-tabulations were performed to explore associations between categorical variables. The Chi-square test of independence was applied to determine whether a statistically significant relationship existed between variables. When significant associations were found, symmetric measures (such as Phi, Cramer’s V, and Contingency Coefficient) were reported to assess the strength and direction of the relationship.

Statistical significance was set at *p* < 0.05, indicating statistically significant differences or associations between the groups or variables tested.

## 3. Results

Table 1 and Table 2 summarize the characteristics of the participants. The CI group included n = 103 children, with a balanced distribution across genders. Most children received bilateral implants (n = 85, 82.5%). The mean age at switch-on was 23.5 months (SD = 8.6). A total of 38.8% (n = 40) had ADs, most commonly developmental delay (n = 14, 30%).

The cohort participated in a single assessment with the PPVT-5, with an average chronological age of 7 years 6 months (SD = 2.8) and an average hearing age of 5 years 5 months (SD = 2.5).

**Figure 1:**
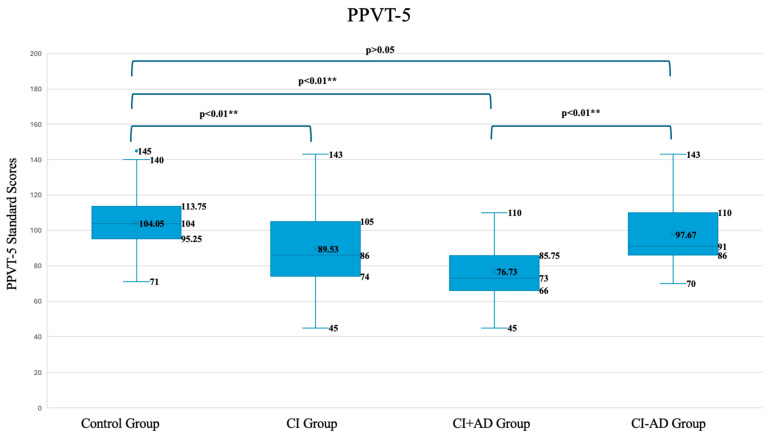
PPVT-5 standard scores of children with cochlear implants, with and without additional difficulties, compared to typically hearing controls. Box plots illustrate score distribution across subgroups; dots represent outliers. ** indicates statistical significance at *p* < 0.01. The mean standard score per American norms is 100 (SD = 15). PPVT-5: Peabody Picture Vocabulary Test 5th edition. CI group: all children with cochlear implants. CI − AD: Children with cochlear implants without additional difficulties. CI + AD: Children with cochlear implants with additional difficulties.

Figure 1 presents the scores of the PPVT-5 in box plots across the study groups, showing the median as a horizontal line and mean scores represented by an X. The boxes indicate the interquartile range (IQR). Whiskers represent the spread of data, and individual points beyond the whiskers show outliers. The mean score for children with TH (M = 104.04, SD = 16.75) was significantly higher than that of the overall children with CI (M = 89.53, SD = 20.51, *p* = 0.00, *p* < 0.01 **). However, both groups scored within the normative range. When divided into subgroups, children with TH performed slightly higher (M = 104.04, SD = 16.75) than those with CI − AD (M = 97.67, SD = 18.81), although the difference was not statistically significant (*p* = 0.069). On the other hand, the CI + AD had markedly lower scores (M = 76.73, SD = 14.99), significantly poorer than CI − AD (*p* = 0.00, *p* < 0.01 **).

Within the CI group, no significant differences in PPVT-5 scores were observed based on maternal education (*p* = 0.32), paternal education (*p* = 0.62), maternal employment (*p* = 0.187), or consanguinity (*p* = 0.69), all *p* > 0.05. In contrast, the presence of ADs showed a significant difference with *p* = 0.000. Age at switch-on was also significantly related to outcomes (*p* = 0.000), with a negative association indicating that later implantation was linked to lower PPVT-5 scores (Γ = –437, *p* = 0.000). The strength of this relationship was moderate.

**Figure 2:**
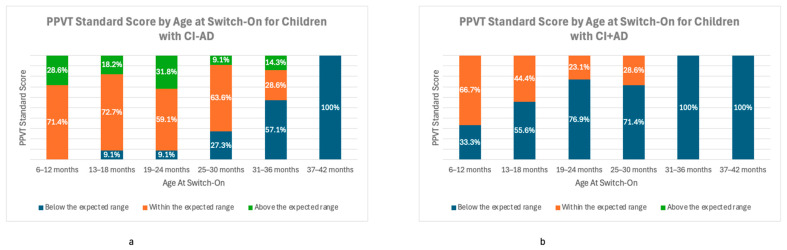
Distribution of PPVT-5 score categories by age at switch-on. (**a**) Children with cochlear implants without additional difficulties (CI − AD). (**b**) Children with cochlear implants with additional difficulties (CI + AD). Scores are categorized as below expected (blue), within expected (orange), and above expected (green).

Figure 2 shows the distribution of PPVT-5 scores across age at switch-on groups for children CI − AD (Figure 2a) and CI + AD (Figure 2b). For children CI − AD, most of those implanted early between 6 and 24 months scored within the expected range (n = 26, 65%), and several achieved scores above the expected range (n = 11, 27.5%). A minority, particularly those outside the 6–12 m group, fell below the expected range (n = 3, 8%). In contrast, later switch-on above 25 m was associated with a higher number of children performing below expected (n = 11, 48%), and a considerable portion remained in the expected range (n = 9, 39%). For CI + AD Figure 2b, outcomes were generally lower across all age groups at switch-on. Among those implanted early (6–24m), only 9 (36%) scored within the expected range, while the remaining 16 (64%) were below expected. In the later switch-on groups implanted older than 25 m, the distribution shifted more: only 2 (13%) reached a score within the expected range, and the remaining 13 (87%) were below expectation. Table 3 clarifies the number of children in each group, as shown in Figure 2, by cross-tabulating age at switch-on groups with their hearing age at the time of the assessment.

## 4. Discussion

This multi-centered, cross-sectional comparative study focused on receptive vocabulary among children with CI from the GCC and compared them to a control group with typical hearing from the same region and to the norm from the PPVT-5. We also examined the factors affecting receptive language, grouping children with CI based on the presence (CI + AD) or absence (CI − AD) of other disabilities.

The mean standard score for children with CI (M = 89.53) was significantly lower than that of their TH peers (M = 104.04), although both mean scores were within the normative ranges. These findings are consistent with the previous literature showing that children with CI tend to achieve receptive vocabulary levels within the normative range, although often lower than their TH peers. Busch et al. found that CI users had lower levels of receptive vocabulary compared to their TH peers (M = 84.6 vs. 102.1), with mean scores around one standard deviation below the population mean [14]. Percy-Smith et al. reported that children with HL achieved receptive vocabulary scores within the normative range, although their TH peers still achieved higher mean scores [19]. Wischmann et al. confirmed in their study that their cohort of children had a mean PPVT score (M = 96) within the norm [32]. In the case of Arab children, limited research has focused on receptive language, and available studies show that most children lag behind. El-Horbety et al. studied a cohort of children with CI with a mean age of 7 years and 7 months and found that their receptive language mean age was only 2.9 years (±1.35), indicating an average receptive language delay of nearly 4 to 5 years compared to their chronological age [33]. It is important to note that the researchers mainly examined global language ability rather than receptive language alone, revealing that 56.5% of participants were delayed compared to the norm, and 43.5% achieved age-appropriate scores. It was not mentioned whether any of those children had ADs. Their cohort, though Arabic-speaking, differed from ours in region and participant characteristics, and the author did not specify whether any children had ADs. Their mean age at implantation was 4.1 years, which is significantly later than our group, for which the mean age at implantation is 23.5 months and no child was implanted later than at 3.5 years of age.

When we analyzed CI − AD children separately, there were no significant difference between their mean receptive vocabulary and that of their TH peers (M = 97.67 vs. M = 104.04). Hardman et al. discussed, through three contrasting case studies, that children with CI who have positive supporting factors and no additional neurological or developmental issues can follow developmental patterns observed in TH children [34]. In their study of Arabic-speaking children from Egypt who do not have ADs and who have unilateral CIs, Farag et al. found, contrary to our findings, that receptive language skills remained markedly below those of typically hearing peers [35]. However, their cohort was implanted at a later age, and there was no information about the type of post-implant therapy used. Studies have shown that children in AVT, like our cohort, develop a higher level of listening and spoken language than those in more traditional types of therapy [36,37], achieving levels comparable to TH children [38].

Our group of children with CI + AD had a lower receptive vocabulary score than those with CI − AD and TH children: the existence of cognitive, developmental, or motor comorbidities (e.g., Autism Spectrum Disorder, cognitive delay, cerebral palsy…) is thus associated with less favorable language outcomes in children with HL [25]. Clinical research on the benefit of CI for this group has indeed shown that these children tend to perform below their peers, even when controlling for factors such as age at implantation and exposure to therapy [25]. In Denmark, for instance, children with other disabilities have shown positive development in receptive language through 6 years of follow-up, but they were still performing poorer than their peers without AD [19]. The combined challenge of HL paired with ADs is a critical obstacle to meeting standard linguistic milestones [39], especially when service provision systems are not well integrated, as is largely the case in the Arab world [40]. Within our CI + AD group, the range was broad, with some children performing better than others, which indicates that the type, severity, and management of comorbidities influence outcomes, as discussed by Cupples et al., who emphasize the severity of having ADs [41]. However, given the overlap between HL and certain developmental profiles, it is not always possible to fully disentangle their respective contributions to the observed language outcomes. To illustrate, some children with mild developmental delays and learning difficulties have shown greater progress in receptive vocabulary than those with Autism Spectrum Disorder (ASD). Given the heterogeneity of our CI + AD group, it is important not to generalize too broadly. Furthermore, it is crucial to respect that language development for this group may take longer.

One of the factors most strongly associated with outcomes in our data was earlier age at switch-on. Children implanted at younger ages tended to have higher PPVT-5 scores. For both groups, CI − AD and CI + AD, earlier implantation was associated with better outcomes. Children with CI − AD implanted before 12 months of age were all within or above the expected normative range. For children implanted between 12 and 24 months, only 8% (n = 3) fell into the below-expected category. For those implanted after 25 months, 52% performed below expectations (n = 12). Notably, all five children implanted after 37 months were below the expected range. We observed a similar pattern for children with CI + AD: 36% (n = 9) scored within the expected range when implanted before 24 months. In the later switch-on groups implanted after 25 months, only 13% (n = 2) scored within the expected range. These findings should be interpreted cautiously, as small group sizes, the type of ADs, and other underlying factors related to delayed diagnosis and implantation may influence language outcomes.

Our finding aligns with the extensive literature on auditory sensitive periods: earlier implantation is associated with more favorable language outcomes [13,42,43]. Karltorp emphasized better results when implantation is done before the age of 9 months [17]. Neurobiologically, earlier auditory access supports more normative cortical development, better synaptic pruning, and more efficient formation of phonological and lexical networks. Delayed implantation risks cross-modal plasticity, where the auditory cortex is recruited for visual or somatosensory processing, diminishing the capacity for subsequent auditory-linguistic mapping [44]. In the GCC, delayed diagnoses and stigma around HL often result in intervention being sought beyond the optimal age [27], which correlates with generally poorer language outcomes, including receptive vocabulary.

Additional disabilities emerge as a strong negative influence. Research indicates that children with additional disabilities, such as cognitive or neurodevelopmental comorbidities, may not achieve the expected language outcomes but do show improvements in quality of life [45,46]. Despite these findings, very few studies look at children with CI + AD [47]. In Arab countries specifically, this group is commonly overlooked. In Saudi Arabia, Mesallam et al. (2019) studied CI + AD children and used questionnaires to compare their results to those of CI − AD children, finding a significant difference [48]. Our findings are similar, although some children (28%) still achieve comparable results to their CI − AD peers (n = 11), especially when implantation is done early [43]. All children who exhibited comparable results were implanted at earlier ages, and the type of difficulty also impacts their results [41]. This subgroup is heterogeneous, comprising a range of developmental conditions with varying types and severities, which likely contributes to the observed variability in outcomes. These findings should therefore be interpreted with caution, as language development in this population may follow a longer and more variable trajectory. While different approaches may be considered in clinical practice depending on individual needs, the present study focuses on spoken language outcomes within a listening and spoken language framework. Future studies using larger samples and multivariate approaches may help clarify the relative contribution of these factors.

Other factors, such as HA use before implantation and parental educational level, had no significant connection to the PPVT results. As in our previous research, these two factors showed no impact on outcomes. Prolonged use of HA prior to cochlear implantation may delay implantation and has no positive effect on receptive vocabulary after cochlear implantation. In terms of parental education, we found no effect on receptive vocabulary, contradicting many earlier studies showing maternal education to be strongly linked to all language outcomes [49]. Studies from Scandinavian countries like Norway and Denmark, as well as the LOCHI study, found a limited association between parental education and vocabulary/language development [14,50,51].

## 5. Limitations

First, our CI + AD subgroup was relatively small and very diverse, which means that the results of this group should be interpreted cautiously. In addition, detailed information on the diagnostic process for ADs was not available, and potential overlap between HL and certain developmental conditions cannot be excluded. Second, the cross-sectional design limits our ability to observe vocabulary growth trajectories over time; a longitudinal design would enable stronger causal inference and modeling of growth rates. Third, the PPVT-5, although widely used, is standardized for normative English-speaking populations. The use of an Arabic-adapted version without locally established normative data may introduce cultural or linguistic biases and should therefore be interpreted cautiously. However, the inclusion of a large control group of Arab children with TH scored according to the American norm provided contextual reference and supported the interpretability of the findings. Finally, details regarding the frequency and duration of AVT were not collected and may represent an additional factor influencing variability in outcomes.

## 6. Conclusions

In conclusion, our investigation shows that a pediatric population with CI in the GCC presents with a delay in receptive vocabulary levels compared to TH peers. Factors such as age at switch-on and the presence of additional difficulties were associated with differences in outcomes. Children with CI − AD demonstrated receptive vocabulary levels comparable to TH peers, while those with comorbidities showed more variable and generally poorer outcomes, and therefore continue to face challenges. The findings emphasize the importance of early screening and cochlear implantation, as well as individualized auditory-verbal therapy tailored to patient profiles. In the future, researchers should consider longitudinal designs, evidence-based Arabic-normed vocabulary, and expanded subgroup sample sizes.

## Figures and Tables

**Table 1:** Baseline characteristics of the study participants.

Variables	Children with CI		CI − AD		CI + AD	
	n	Percent	n	Percent	n	Percent
**Demographic** **Characteristics**						
**Country**						
Bahrain	24	23.3%	16	25.4%	8	20.0%
KSA ^1^	69	67.0%	43	68.3%	26	65.0%
Kuwait	5	4.9%	3	4.8%	2	5.0%
UAE ^2^	5	4.9%	1	1.6%	4	10.0%
**Gender**						
Girl	56	54.4%	34	54.0%	22	55.0%
Boy	47	45.6%	29	46.0%	18	45.0%
**Clinical** **C** **haracteristics**						
**Use of HA prior to CI**						
No	24	23.3%	15	23.8%	9	22.5%
Yes	79	76.7%	48	76.2%	31	77.5%
**Site of the implant**						
Bilateral	85	82.5%	53	84.1%	32	80.0%
Unilateral	18	17.5%	10	15.9%	8	20.0%
**Age at switch** **-** **on in months**						
6–12 months	10	9.8%	7	11.1%	3	7.7%
13–18 months	20	19.6%	11	17.5%	9	23.1%
19–24 months	35	34.3%	22	34.9%	13	33.3%
25–30 months	17	16.7%	11	17.5%	6	15.4%
31–36 months	12	11.8%	7	11.1%	5	12.8%
37–42 months	8	7.8%	5	7.9%	3	7.7%
**Etiology if known**						
Acquired	7	6.8%	2	3.2%	5	12.5%
Genetics	40	38.8%	25	39.7%	15	37.5%
Unknown	56	54.4%	36	57.1%	20	50.0%
**Family** **Characteristics**						
**Maternal Employment**						
No	76	73.8%	42	66.7%	34	85.0%
Yes	27	26.2%	21	33.3%	6	15.0%
**Maternal Education**						
None	2	2.0%			2	5.0%
School	46	45.1%	24	38.7%	22	55.0%
Diploma	3	2.9%	2	3.2%	1	2.5%
University	51	50.0%	36	58.1%	15	37.5%
**Paternal Education**						
None	1	1.0%			1	2.6%
School	39	38.6%	22	35.5%	17	43.6%
Diploma	3	3.0%	2	3.2%	1	2.6%
University	58	57.4%	38	61.3%	20	51.3%
**Consanguinity**						
No	28	27.2%	17	27.0%	11	27.5%
Yes	75	72.8%	46	73.0%	29	72.5%
**Additional** **Difficulties**						
CI − AD ^3^ (Without)	63	61.2%			63	100.0%
CI + AD ^4^ (With)	40	38.8%	40	100.0%		
**Type of Additional** **Difficulties**						
Cognitive Delay	6	15.0%			6	0.2%
Developmental Delay	14	35.0%			14	0.4%
Cerebral Palsy	7	17.5%			7	0.2%
Learning Difficulties	4	10.0%			4	0.1%
Vision Problem	3	7.5%			3	0.1%
Hypotonia	2	5.0%			2	0.1%
Apraxia	1	2.5%			1	0.0%
ASD ^5–7^	2	5.0%			2	0.1%
ADHD ^6^	5	12.5%			5	0.1%
Down Syndrome ^7^	1	2.5%			1	0.0%
Epilepsy	2	5.0%			2	0.1%

^1^ KSA: Kingdom of Saudi Arabia. ^2^ UAE: United Arab Emirates, here referring only to Dubai. ^3^ CI − AD: Children with cochlear implants without additional difficulties. ^4^ CI + AD: Children with cochlear implants with additional difficulties. ^5^ ASD: Autism Spectrum Disorder. ^6^ ADHD: Attention Deficit Hyperactivity Disorder. ^7^ Children diagnosed with Down syndrome or ASD could present with multiple difficulties; however, each was categorized according to the primary diagnosis recorded in medical charts.

**Table 2:** Age-related characteristics of the participant groups.

Variables	Children with CI ^1^			CI − AD ^2^			CI + AD ^3^		
	n	Mean	SD	n	Mean	SD	n	Mean	SD
**Age at Diagnosis in months**	103	8.78	8.69	63	9.27	8.58	40	8.00	8.91
**Age at switch** **-** **on in months**	103	23.53	8.63	63	23.29	8.23	40	23.93	9.32
**Chronological age in years**	103	7.65	2.80	63	7.70	2.96	40	7.58	2.55
**Hearing age in years**	103	5.51	2.52	63	5.62	2.72	40	5.33	2.20

^1^ CI: cochlear implants. ^2^ CI − AD: Children with cochlear implants without additional difficulties. ^3^ CI + AD: Children with cochlear implants with additional difficulties.

**Table 3:** Cross-tabulation of age at switch-on and hearing age at the time of assessment.

Hearing Age in Years Switch-On Age in Months	2–3 y	3–4 y	4–5 y	5–6 y	6–7 y	7–8 y	8–9 y	9–10 y	Total
6–12 m	2	3	1	1	0	2	1	0	20
13–18 m	4	4	3	5	3	1	0	0	35
19–24 m	3	3	6	6	6	6	2	3	17
25–30 m	2	7	1	2	0	2	2	1	12
31–36 m	3	0	4	0	1	1	1	2	9
37–42 m	1	1	2	1	0	2	1	1	10
Total	15	18	17	15	10	14	7	7	103

## Data Availability

Data are unavailable due to privacy restrictions, as participants have not provided consent to share their data with individuals not directly involved in this study.

## Abbreviations

The following abbreviations are used in this manuscript:

CIs	Cochlear Implants
TH	Typical hearing
AVT	Auditory-verbal therapy
HL	Hearing loss
GCC	Gulf Cooperation Council
PPVT-5	Peabody Picture Vocabulary Test 5th edition
ADs	additional difficulties
KSA	Kingdom of Saudi Arabia
UAE	United Arab Emirates, here referring only to Dubai
CI − AD	cochlear implants without additional difficulties.
CI + AD	Cochlear implants and additional difficulties.
ASD	Autism Spectrum Disorder
ADHD	Attention Deficit Hyperactivity Disorder
